# Cardiac Subtype-Specific Modeling of K_v_1.5 Ion Channel Deficiency Using Human Pluripotent Stem Cells

**DOI:** 10.3389/fphys.2017.00469

**Published:** 2017-07-06

**Authors:** Maike Marczenke, Ilaria Piccini, Isabella Mengarelli, Jakob Fell, Albrecht Röpke, Guiscard Seebohm, Arie O. Verkerk, Boris Greber

**Affiliations:** ^1^Human Stem Cell Pluripotency Laboratory, Max Planck Institute for Molecular BiomedicineMünster, Germany; ^2^Chemical Genomics Centre of the Max Planck SocietyDortmund, Germany; ^3^Department of Cardiovascular Medicine, Institute of Genetics of Heart Diseases, University of Münster Medical SchoolMünster, Germany; ^4^Department of Clinical and Experimental Cardiology, Academic Medical Center, University of AmsterdamAmsterdam, Netherlands; ^5^Institute of Human Genetics, University of MünsterMünster, Germany; ^6^Department of Medical Biology, Academic Medical Center, University of AmsterdamAmsterdam, Netherlands

**Keywords:** induced pluripotent stem cells, disease modeling, cardiac differentiation, K_v_1.5, atrial fibrillation

## Abstract

The ultrarapid delayed rectifier K^+^ current (I_Kur_), mediated by K_v_1.5 channels, constitutes a key component of the atrial action potential. Functional mutations in the underlying *KCNA5* gene have been shown to cause hereditary forms of atrial fibrillation (AF). Here, we combine targeted genetic engineering with cardiac subtype-specific differentiation of human induced pluripotent stem cells (hiPSCs) to explore the role of K_v_1.5 in atrial hiPSC-cardiomyocytes. CRISPR/Cas9-mediated mutagenesis of integration-free hiPSCs was employed to generate a functional *KCNA5* knockout. This model as well as isogenic wild-type control hiPSCs could selectively be differentiated into ventricular or atrial cardiomyocytes at high efficiency, based on the specific manipulation of retinoic acid signaling. Investigation of electrophysiological properties in K_v_1.5-deficient cardiomyocytes compared to isogenic controls revealed a strictly atrial-specific disease phentoype, characterized by cardiac subtype-specific field and action potential prolongation and loss of 4-aminopyridine sensitivity. Atrial K_v_1.5-deficient cardiomyocytes did not show signs of arrhythmia under adrenergic stress conditions or upon inhibiting additional types of K^+^ current. Exposure of bulk cultures to carbachol lowered beating frequencies and promoted chaotic spontaneous beating in a stochastic manner. Low-frequency, electrical stimulation in single cells caused atrial and mutant-specific early afterdepolarizations, linking the loss of *KCNA5* function to a putative trigger mechanism in familial AF. These results clarify for the first time the role of K_v_1.5 in atrial hiPSC-cardiomyocytes and demonstrate the feasibility of cardiac subtype-specific disease modeling using engineered hiPSCs.

## Introduction

Atrial fibrillation (AF) constitutes the most prevalent cardiac arrhythmia and accounts for about one third of all hospitalizations related to heart rhythm disturbances (Fuster et al., 2011). Although not lethal *per se*, persistent uncoordinated atrial activation may lead to remodeling and deterioration of atrial function, which is associated with a reduced quality of life and increased long-term risk of stroke and overall mortality (Wolf et al., 1991). The prevalence of AF is in the range of 1% in the general population and tightly increasing with age, thereby imposing a substantial and steadily rising burden on society (Kannel and Benjamin, 2008). Current treatment options are limited, however, and mostly centered around controlling risk scores for stroke and adverse symptoms associated with AF (Fabritz et al., 2016).

AF frequently develops secondary to other conditions such as hypertension and other cardiovascular diseases (Schotten et al., 2011). However, a substantial proportion of AF patients displays no history of AF-associated disorders, which also suggests a hereditary component (lone AF) (Fuster et al., 2011). Indeed, a familial history of AF has been associated with a 40% risk increase for developing it and over the last years, a number of AF-related gene loci and mutations have been identified (Lubitz et al., 2010; Hucker et al., 2016). These comprise several developmental regulators such as *PITX2* as well as ion channel genes involved in cardiac action potential (AP) generation, suggesting a direct causative role of the latter group (Kirchhof et al., 2011; Christophersen and Ellinor, 2016).

Channelopathies in particular are amenable to disease modeling using patient-derived or genetically engineered human induced pluripotent stem cells (hiPSCs), as they tend to mediate relevant phenotypes in a cell-automomous manner (Zhang et al., 2014; Bezzerides et al., 2016; Malan et al., 2016). Hence, despite the fact that AF penetrance and progression is undoubtedly linked to the organismal context, hiPSC-based AF models may allow for investigating disease phenotypes and drug responses at the cellular and tissue levels. However, most hiPSC-derived cardiomyocytes (CMs) tend to acquire a ventricular rather than an atrial fate, which has until now hampered efforts to explore aspects of AF using the hiPSC system. Interestingly, this methodological hurdle has recently been overcome through manipulation of retinoic acid (RA) signaling, a physiological pathway that also drives atrial specification *in vivo* (Zaffran et al., 2014; Devalla et al., 2015).

Given these recent technical advances, we here seek to explore the possibility of cardiac subtype-specific disease modeling with potential relevance to familial AF by focussing on the atrial-specific K^+^ channel K_v_1.5 (encoded by *KCNA5*). K_v_1.5 conducts the ultrarapid delayed rectifier K^+^ current (I_Kur_) (Schmitt et al., 2014). Interestingly, both loss and gain-of-function mutations in *KCNA5* have been shown to cause familial AF (Olson et al., 2006; Yang et al., 2009; Christophersen et al., 2013; Hayashi et al., 2015). This seeming paradox is explained by the fact that AF may be promoted via distinct mechanisms, namely, triggered activity and electrical reentry. While the latter tends to be favored by AP shortening, the likelihood for the former becomes increased by AP prolongation. Hence, in agreement with the widely accepted role of K_v_1.5 in the repolarization of atrial CMs, a corresponding knockout model would be based on that former rationale (Schmitt et al., 2014). Indeed, QTc interval prolongation predisposes for arrhythmia-inducing triggering events such as early afterdepolarizations (EADs) and is considered a risk factor in particular for lone AF cohorts (Johnson et al., 2008; Nielsen et al., 2013).

In this context, we here report an isogenic *KCNA5* knockout model in hiPSCs-CMs, to investigate basic as well drug-induced phenotypes in a cardiac subtype-specific manner.

## Materials and methods

### Generation, characterization, and genetic engineering of hiPSCs

Wild-type hiPSCs were derived from fibroblasts of pooled fetal livers originally isolated in 2006 (a gift by Dr. Tobias Cantz, Hannover Medical School). These were expanded in 10% serum/Knockout™ DMEM (Thermo)/1 x penicillin/streptomycin/glutamine (PSG, Thermo), replated at 100,000 cells per well of a 12-well plate, and transfected next day using 4 μl Ribojuice (Merck) and 1 μg of self-replicating RNA containing reading frames for OCT4, SOX2, KLF4, and GLIS1 (Addgene plasmid #58974). Reprogramming RNA was generated as previously described (Yoshioka et al., 2013), using a RiboMAX T7 *in vitro* transcription kit (Promega) on linearized plasmid DNA, and Cellscript kits #C-SCCE0610, #C-SCMT0610, and #CPAP5104H for RNA capping, 2′-O-Methylation, and PolyA tailing, respectively. Reprogramming medium contained 100 ng/ml B18R protein (eBioscience), 0.5 μg/ml puromycin (for 3 days, then 0.1 μg/ml), 0.5 mM valproic acid, and 5 μM EPZ004777 (Tocris). hPSC-like colonies emerging several weeks later were picked and expanded under hiPSC maintenance conditions (see below). A selected, newly generated hiPSC line termed F1 was characterized using a panel of assays which have been described elsewhere (Greber et al., 2011). Karyotyping was performed according to standard procedures (*n* = 10). Primers for RT-qPCR and bisulfite sequencing are given in Table S2.

**Figure 1:**
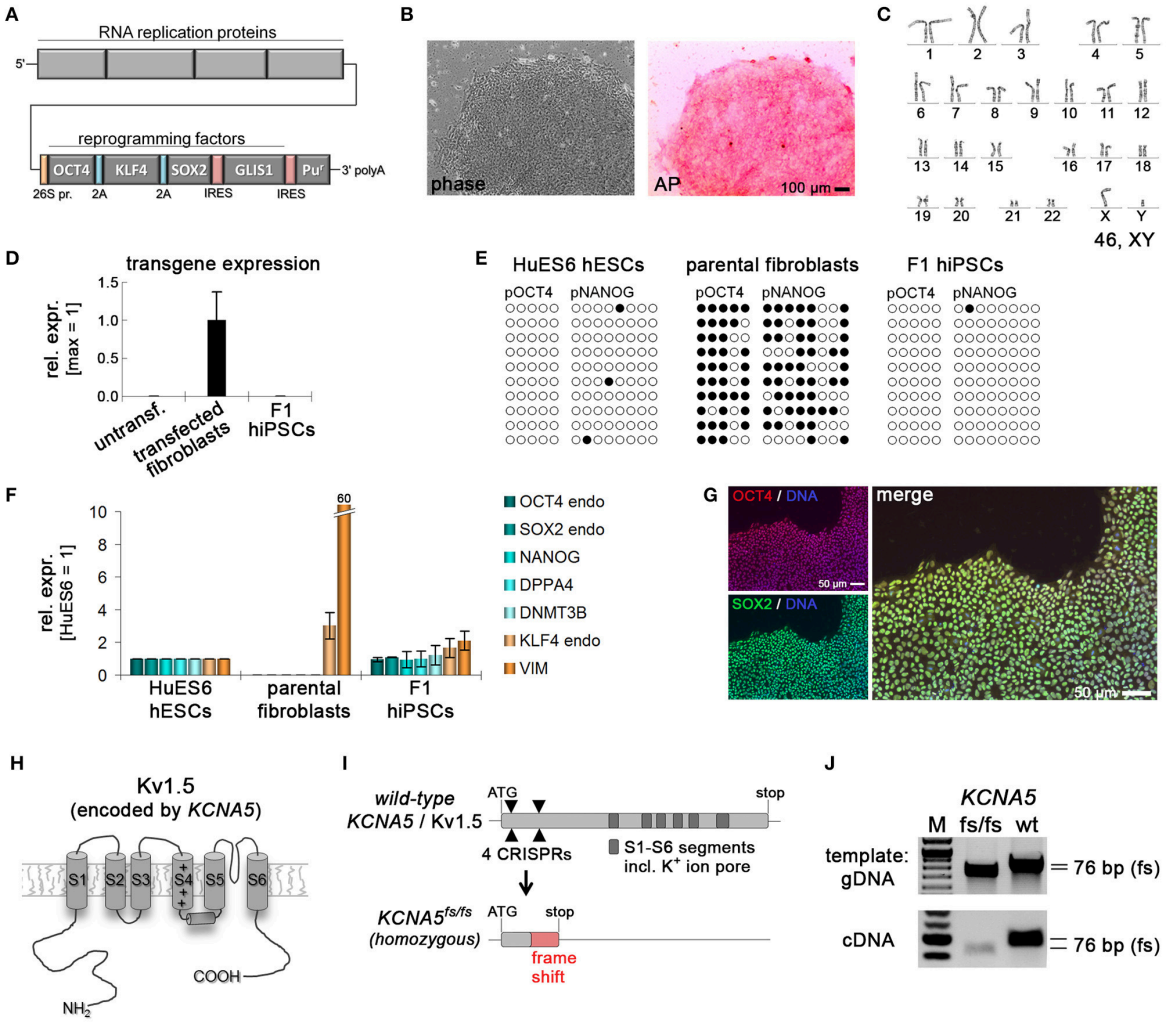
Generation of *KCNA5* knockout model. **(A)** Illustration of polycistronic self-replicating RNA used for reprogramming (Yoshioka et al., 2013). Reading frames for OCT4, KLF4, SOX2, GLIS1, and puromycin resistance are separated by 2A peptide-encoding or IRES sequences. **(B)** Phase contrast morphology (5 x) and alkaline phosphatase staining of the F1 wild-type hiPSC line generated using the RNA vector shown in **(A)**. **(C)** Representative F1 karyogram indicating a normal male genotype. **(D)** The RNA vector is undetectable in established F1 hiPSCs following puromycin withdrawal, whereas freshly transfected parental fibroblasts display robust transgene expression (RT-qPCR data, *n* = 2). **(E)** Bisulfite sequencing of OCT4 and NANOG promoter regions of the indicated samples showing an hESC-like hypomethylated state in F1 cells. **(F)** RT-qPCR analysis of F1 hiPSCs in comparison to the original fibroblasts and a hESC control (*n* = 2). **(G)** Immunostains showing robust pluripotency factor expression in F1 hiPSCs at the protein level. **(H)** Illustration of the K_v_1.5 channel. The voltage sensor in transmembrane helix S4 is indicated by symbols. The ion pore region is in-between segments S5 and S6. **(I)** *KCNA5* knockout strategy based on simultaneously transfecting 4 CRISPR/Cas9 nickase vectors targeting the 5' end of the 1-exon gene body. A mutant clone generated this way displayed homozygous deletions causing a frame shift and premature stop as illustrated at the bottom. **(J)** PCR spanning the mutation site validates the sequencing-verified frame shift-causing deletion in KCNA5^fs/fs^ cells at the DNA and RNA levels.

For *KCNA5* mutagenesis, a “4n” CRISPR approach targeting the 5′ end of the *KCNA5* coding sequence was employed, using *4* CRISPR/Cas9*n* nickase vectors as illustrated in Figure 1I. Fwd oligonucleotide sequences defining the gRNAs targeting this genomic region are given in Table S2. These were phosphorylated, annealed with their corresponding reverse oligos and cloned into a modified version of pX335 (Addgene plasmid #42335) carrying a GFP-puromycin selection cassette. F1 hiPSCs were co-transfected with all 4 CRISPR vectors using Fugene HD (Roche) and transiently selected for 24 h using 0.5 μg/ml puromycin, to enrich for transfected cells. Following replating of the cells at clonal dilution several days after transfection, clonal half-colonies were picked another ~2 weeks later, to be analyzed for successful mutagenesis. This was done using conventional DNA isolation and PCR spanning the target region (Table S2). CRISPR-induced genomic deletions were indicated by smaller PCR products which were subsequently cloned and sequenced (~10 TOPO clones per mutant hiPSC line, to statistically obtain sequence information on both alleles).

### Maintenance of hiPSCs

F1 hiPSCs, the KCNA5^fs/fs^ derivative line, and HuES6 human embryonic stem cells were routinely cultured in 6-well plates on 1:75 diluted Matrigel™ HC (Corning #354263), in FTDA medium [10]. FTDA consisted of DMEM/F12, 1 × ITS (Corning #354350), 0.1% human serum albumin (Biological Industries #05-720-1B), 1 × defined lipids (all Thermo), 1 × PSG, 10 ng/ml FGF2 (PeproTech #100-18B), 0.2 ng/ml TGFβ1 (eBioscience #34-8348-82), 50 nM Dorsomorphin (Santa Cruz #sc-200689), and 5 ng/ml Activin A (eBioscience #34-8993-85). Cells were routinely passaged as single cells or, initially, as clumps of cells. For single cell splitting, cells were grown to full confluence (until cultures seemingly appeared syncytial), digested for 15 min using Accutase™ containing 10 μM Y-27632 (R&D # 1254/50), and replated in the presence of Y-27632 at 600,000 cells per well of a 6-well plate. hiPSCs reached full confluence after about 4 days under these conditions and were subsequently harvested as above, for continuous maintenance or for the induction of differentiation. hiPSCs were kept in culture for a maximum of 30 passages. Cell lines were tested negative for mycoplasma.

### Cardiac differentiation

Cardiac induction was performed under serum and serum albumin-free conditions (Zhang et al., 2015). Fully confluent hiPSC cultures—which then appeared as a flat, seemingly syncytial monolayer—were harvested using Accutase™, centrifuged twice at 300 g, resuspended in day 0 differentiation medium and seeded out at 550,000 cells per well of a Matrigel-coated 24-well plate, in a total volume of 2 ml. Compared to maintenance plates, differentiation plates were coated with a further 1:3 dilution of Matrigel (a total dilution of approximately 1:250). Day 0 differentiation medium consisted of KO-DMEM, 1 × ITS, 10 μM Y-27632, PSG, 5 ng/ml Activin A, 10 ng/ml FGF2, 0.5–1 ng/ml BMP4 (R&D #314-BP-050), and 1 μM CHIR99021 (AxonMedchem #Axon 1386). Before placing freshly seeded differentiation cultures back into the incubator, plates were tapped several times under a stereo microscope and then left standing at room temperature for 30 min, to ensure an even distribution and initial attachment of cells. Medium in differentiation plates was exchanged on a daily basis. From day 1 onwards, the basal differentiation medium consisted of KO-DMEM, 1 × TS (transferrin/selenium), 250 μM 2-phospho-ascorbate (Sigma-Aldrich #49752), and PSG. 100 × TS stock was prepared in advance by dissolving 55 mg transferrin (Sigma #T8158) in 100 ml PBS containing 0.067 mg sodium selenite (Sigma #S5261). WNT inhibitor C-59 (Tocris #5148/10) was added to the cultures from 48to 96 h of differentiation at 0.2 μM, to promote cardiac specification. Optionally, for promoting an atrial fate, all-trans-retinoic acid (Sigma #R2625-50MG) was supplemented from 72 to 120 h of differentiation.

Differentiating cultures typically started to display spontaneous beating after 6 (no RA) or 8 days (+RA). To allow maturation of the cultures until the preferred time-point of analysis (typically at ~2.5 weeks after the initiation of differentiation), beating monolayers were dissociated using TrypLE Select (Thermo) and replated at a ratio of ~1:4 using CM splitting medium consisting of RPMI 1640 (Thermo), 1 × ITS, 0.2% (w/v) HSA, 250 μM phospho-ascorbate, 0.008% thioglycerol, PSG, and 10 μM Y-27632. Next day, medium was replaced by CM maintenance medium consisting of KO-DMEM, 1 × ITS, 0.2% (w/v) HSA, 250 μM phospho-ascorbate, 0.008% thioglycerol, and PSG. Following CM maturation, cells were replated once again, at an appropriate ratio, onto MEA or glass surfaces for downstream analyses several days later (~3 week total).

### Electrophysiological analysis on MEAs

For electrophysiological analysis on microelectrode arrays (USB-MEA256 system, Multichannel Systems), the electrode areas of plasma-cleaned 9-well MEAs were coated with 3 μl of a 1:150 diluted Matrigel/0.1% gelatin solution in KO-DMEM for ~2 h at 37°C in a humidified cell culture incubator. hiPSC-CMs were dissociated from maintenance cultures using a 10 × TrypLE Select digestion to obtain a single-cell/small aggregate suspension. Coating solution was removed from the electrode arrays to be replaced by 25,000–50,000 cells resuspended in a ~3 μl droplet of CM replating medium. CMs were allowed to attach for ~30 min. Subsequently, MEA chambers with attached cells were filled with 150 μl of CM replating medium. Next day, medium was replaced by CM maintenance medium once more. From the following day onwards, cell preparations were used for electrophysiological recordings at 37°C. Recordings of drug-treated cells were initiated after a wash-in time of about 5 min (4-AP: 1 mM, propranolol: 10 μM, isoprenaline: 10 μM, AF-DX-116: 10 μM, cisapride: 100 nM, carbachol: 0.2–0.5 mM). Wash-out recordings were performed after five media changes. T_max_ and peak-to-peak finding algorithms were implemented in MC Rack software v4.5.7. Field potential durations (FPDs, QT_max_ intervals) and peak-to-peak (RR) intervals were averaged from five consecutive measurements of a given sample, if appropriate, and averaged between independent replicates. Data were processed in MS Excel where QT_max_-like intervals were frequency-corrected using Bazett's formula: QTc_max_ = QT_max_ [ms]/(RR [s])^0.5^. Poincaré plots were generated from representative recordings.

### Action potential measurements

For action potential (AP) measurements, culture tissues were enzymatically dissociated into single cells as described previously (Meijer van Putten et al., 2015) and plated at a low density on Matrigel-coated coverslips. AP measurements were performed using the amphotericin-B perforated patch-clamp technique and an Axopatch 200B amplifier (Molecular Devices). Data acquisition and analysis were realized with custom software. Signals were low-pass-filtered with a cutoff of 5 kHz and digitized at 40 kHz. The potentials were corrected for the calculated liquid junction potential of 15 mV (Barry and Lynch, 1991). Cell membrane capacitance (C_m_) was determined with -5 mV voltage step from −40 mV by dividing the time constant of the decay of the capacitive transient by the series resistance.

APs were recorded at 36 ± 0.2°C from single and spontaneously contracting hiPSC-CMs. Cells were superfused with solution containing (in mmol/L): 140 NaCl, 5.4 KCl, 1.8 CaCl_2_, 1.0 MgCl_2_, 5.5 glucose, 5.0 HEPES; pH 7.4 (NaOH). Patch pipettes (borosilicate glass; resistance ≈2.5 MΩ) contained (in mmol/L): 125 K-gluconate, 20 KCl, 5 NaCl, 0.44 amphotericin-B, 10 HEPES; pH 7.2 (KOH). hiPSC-CMs typically lack the inward rectifier K^+^ current, *I*_K1_, that limits the functional availability of Na^+^ current (*I*_Na_) and transient outward K^+^ current (*I*_To_) (Hoekstra et al., 2012; Giles and Noble, 2016). To overcome this limitation, we injected an *in silico I*_K1_ with kinetics of Kir_2.1_ channels through dynamic clamp, as we previously described in detail (Meijer van Putten et al., 2015). An amount of 2 pA/pF peak outward current was applied, resulting in quiescent hiPSC-CMs with a maximal membrane depolarization (MDP) of −80 mV or more negative. APs were elicited at 0.2–3 Hz by 3 ms, ~1.2 × threshold current pulses through the patch pipette. We analyzed the MDP, maximum AP amplitude (APA_max_), AP duration at 20, 50, and 90% of repolarization (APD_20_, APD_50_, and APD_90_ respectively), maximal upstroke velocity (V_max_) and plateau amplitude (APA_plat_) measured 20 ms after the AP upstroke. Averages were taken from 10 consecutive APs.

### Immunocytochemistry, FACS, RT-qPCR

Immunofluorescence analysis was carried out according to standard procedures following paraformaldehyde fixation and permeabilization/blocking with 0.2% Triton X-100/5% FCS/2% BSA/2% glycine in PBS for 45 min. Antibodies used were anti-AFP (1:250, Sigma #A8452), ANP (1:50, R&D # AF3366), K_v_1.5 (1:100, Santa Cruz #sc-377110), MLC2v (1:200, ProteinTech Group #10906-1-AP), NKX2.5 (1:200, R&D #AF2444), OCT4 (1:100, Santa Cruz #sc-5279), SMA (1:200, Sigma #C6198), SOX2 (1:200, R&D #AF2018), Troponin C (1:100, Thermo #MS-295-P), Troponin I (1:250, Santa Cruz #sc15368), βIII-Tubulin (1:2000, Covance #PRB-435P), as well as appropriate Alexa Fluor 488 or 568-conjugated secondary antibodies (Thermo). Images shown are full or cropped frames taken with a 10 or 20 × objective mounted to a Zeiss Axiovert inverted microscope.

Intracellular staining prior to FACS analysis was performed as described (Zhang et al., 2015). Briefly, cells were dissociated using TrypLE Select, fixed, and stained in 0.5% saponin/5% FCS in PBS using α-CTNT (1:200, Labvision #MS-295-P) and Alexa-488-conjugated α-mouse (Life Technologies).

RNA was isolated using NucleoSpin RNA kits with on-column DNA digestion (Machery Nagel). Reverse transcription was performed using M-MLV reverse transcriptase (Affymetrix #78306) with oligo-dT_15_ priming at 42°C. Real-time PCR was carried out using validated primers in Table S2 and BioRad iTaq™ Universal SYBR Green Supermix (#172-5124) on an ABI 7500 cycler. Efficiency-validated primers used are given in Table S2. *RPL37A* served as housekeeping control. Data are expressed relative to an indicated control sample or, alternatively, as percentage of RPL37A abundance (100^*^2^−ΔCt^).

### Statistical analysis

Unless otherwise stated, data are presented as means between biological replicates ± SEM. Statistical analysis was carried out with SigmaStat 3.5 software. Two groups were compared using unpaired *t*-tests or, in case of a failed normality and/or equal variance test, Mann-Whitney Rank Sum Test. In action potential measurements, normality and equal variance assumptions were tested with the Kolmogorov-Smirnov and the Levene median test, respectively. More than 2 groups were compared using One-Way ANOVA or Two-Way repeated ANOVA followed by a Student-Newman-Keuls Method *post-hoc* test. In case of non-normally distributed parameters, Kruskal-Wallis test followed by pairwise comparisons with Dunn's Method was performed. *P* < 0.05 defines statistical significance.

## Results

### Generation of a KCNA5 knockout hiPSC model

A previously described approach based on a self-replicating RNA vector expressing four reprogramming factors was employed to generate integration-free wild-type hiPSCs from fetal human fibroblasts (Figure 1A) (Yoshioka et al., 2013). Following RNA vector transfection and antibiotic selection to maintain robust transgene expression (Figure S1A), cell line F1 was obtained and characterized in more detail. F1 hiPSCs showed typical human pluripotent stem cell morphology, stained positive for alkaline phosphatase under hPSC maintenance conditions (Frank et al., 2012), and displayed a normal male karyotype (Figures 1B,C). Following withdrawal of puromycin selection pressure, transgene expression was no longer detectable indicating a transgene-free reprogrammed state, as expected (Figure 1D). Instead, F1 cells showed reset DNA methylation of the *OCT4* and *NANOG* promoters as evidenced by bisulfite sequencing (Figure 1E). Expression of endogenous pluripotency markers was indistinguishable from that in human embryonic stem cells and robustly detectable at protein level (Figures 1F,G).

Immunocytochemical analysis of spontaneously differentiated cultures indicated competency to form derivatives of all three germ layers suggesting acquired pluripotent characteristics (Figure S1B). Finally, a previously established protocol for directed differentiation along the cardiac lineage was applied revealing that CMs could be generated at high efficiency (Figure S1C, Video S1). These data suggest that F1 cells are fully reprogrammed to a pluripotent state.

The *KCNA5* gene encoding K_v_1.5 (Figure 1H) is composed of a single exon. Four CRISPR/Cas9 nickase vectors were designed to target the 5′ end of the open reading frame, to potentially induce frame shift-causing deletion mutations in a controlled manner. Indeed, we were able to isolate one clone showing small homozygous frame shift-causing deletions leading to a premature stop codon on both alleles (Figure 1I). This engineered cell line designated KCNA5^fs/fs^ hence resembles the genotype of a well-documented patient case of lone AF caused by a heterozygous but dominant *KCNA5* nonsense mutation, to lead to a loss of K_v_1.5 function (Olson et al., 2006). The homozygous 76 bp deletion in our model was confirmed using conventional PCR and sequencing at the genomic and transcriptional levels (Figure 1J).

### Cardiac subtype-specific differentiation of WT and KCNA5^fs/fs^ hiPSCs

**Figure 2:**
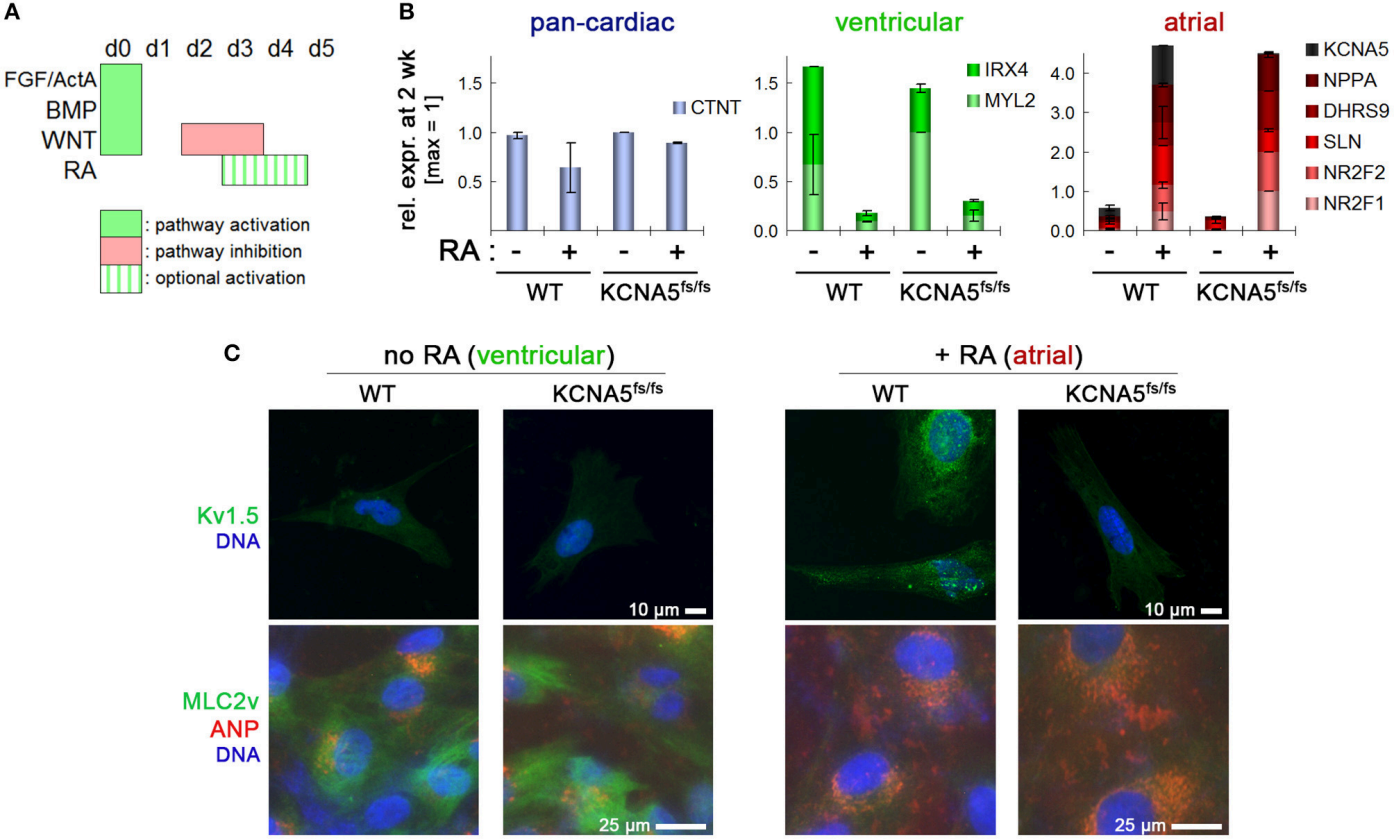
Cardiac subtype-specific differentiation of WT and KCNA5^fs/fs^ hiPSCs. **(A)** Illustration of directed differentiation protocol. Retinoic acid addition (RA, 0.5 μM) during the indicated time interval was used to selectively induce an atrial differentiation fate. **(B)** Subtype-specific cardiac differentiation showing RA-dependent suppression of ventricular gene expression (green) and induction of atrial markers (red) both in WT and KCNA5^fs/fs^ hiPSCs (*n* = 2). Note the diminished KCNA5 expression level in RA-treated mutant cardiomyocytes. **(C)** Immunocytochemical staining of K_v_1.5 (top), atrial natruretic peptide, and ventricular-specific myosin light chain 2 (bottom). Note that the MLC2v staining appears relatively weak and diffuse due to the early time-point of analysis (2.5 week after differentiation start).

Next, we sought to assess whether both the mutant and the wild-type (WT) control lines were capable of undergoing selective differentiation into ventricular vs. atrial-like CMs. The Zhang et al. protocol (Zhang et al., 2015) served as a baseline for ventricular-like differentiation, as this presents the default fate for most cells in virtually any directed differentiation procedure. Optionally, transient stimulation with RA may be administered for conferring an atrial CM identity (Devalla et al., 2015). Optimization experiments suggested that a 2-day supplementation with RA between 72 and 120 h of differentiation presented a sufficient time period (Figure 2A and data not shown). Panels of ventricular and atrial-specific marker genes were defined based on our previous analysis of primary human heart tissue samples in comparison to hPSC-CMs (Piccini et al., 2015). These were used for analyzing RA titration experiments aiming at identifying an optimal dose. Results revealed that a stimulation with 0.5 μM RA during CM induction was sufficient for robustly inducing atrial markers including KCNA5 while strongly suppressing ventricular ones like *IRX4* and *MYL2* (encoding myosin light chain 2, Figure S2A).

Importantly, this variant of the protocol was applicable both to WT and KCNA5^fs/fs^ hiPSCs suggesting that the model as well as its isogenic control may efficiently be converted into the desired cardiac subtype (atrial-enriched CMs). Default ventricular-like CMs could instead serve as an important specificity control in subtype-specific disease modeling (Figure 2B). After ~1.5 weeks of maturation under CM maintenance conditions, RA untreated CMs showed *MYL2* (MLC2v) expression and low atrial natriuretic peptide (ANP) abundance at the protein level, whereas the reverse pattern was obtained after RA treatment (Figure 2C, bottom). By contrast, K_v_1.5 was detectable only in atrial-like WT cells and absent in KCNA5^fs/fs^ CMs, which confirmed the homozygosity of the underlying mutation and the consistency of the differentiation methodology (Figure 2C, Figure S2B).

### The primary disease phenotype in KCNA5^fs/fs^ CMs is atrial-specific

**Figure 3:**
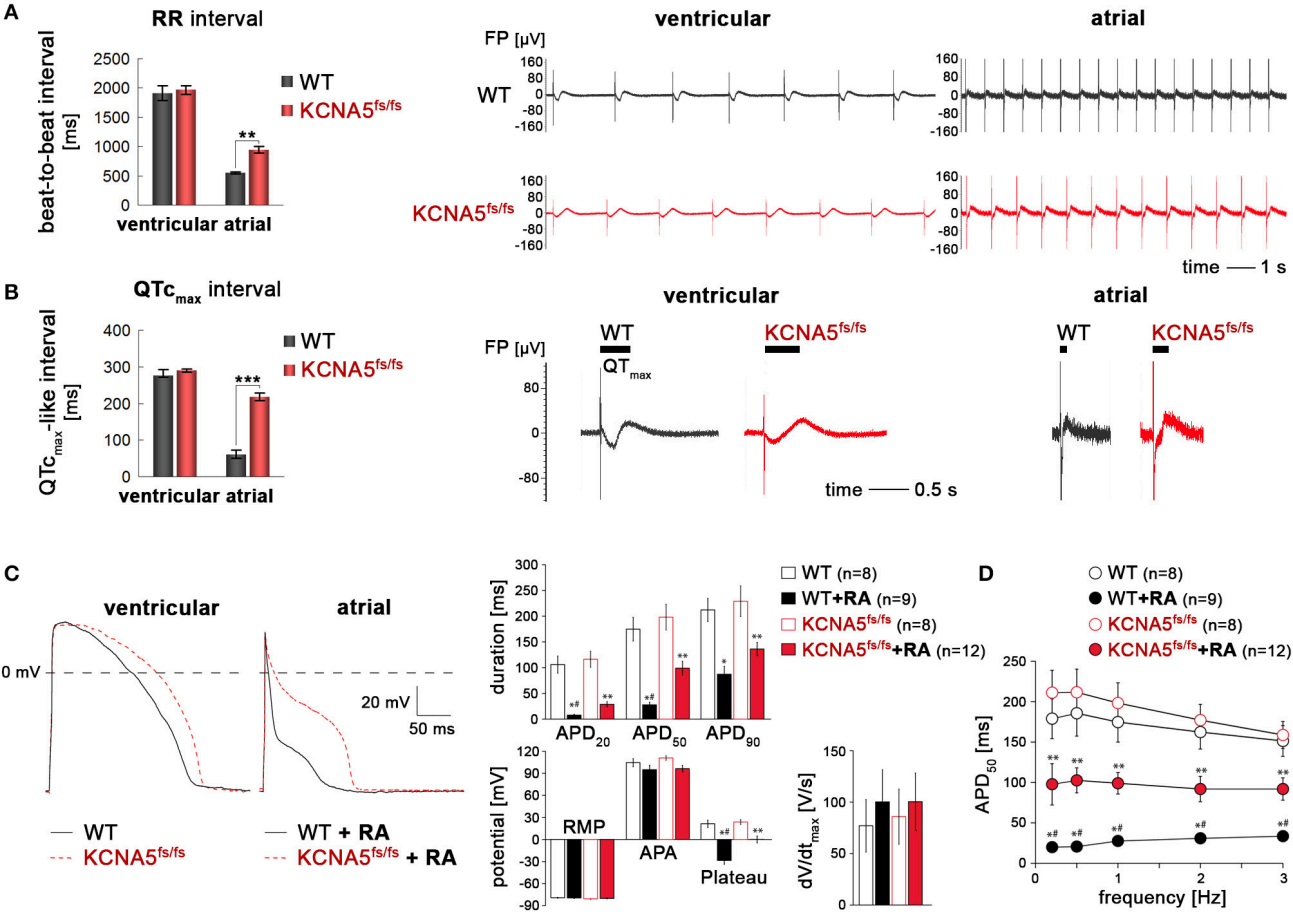
Subtype-specific phenotype of KCNA5^fs/fs^ hiPSC-cardiomyocytes. **(A)** Left: MEA-based quantification of beat-to-beat intervals in ventricular and atrial CMs (*n* = 3, ^**^p < 0.01). Right panels: Representative MEA traces showing frequency differences in spontaneous beating behavior as dependent on genotype and cardiac subtype. **(B)** Frequency-corrected field potential durations in ventricular and atrial WT and KCNA5^fs/fs^ hiPSC-CMs. Quantification from three independent experiments (left, ^***^p < 0.001) and representative MEA traces (right). Horizontal bars indicate QT_max_ intervals which were more accurately determined by averaging several consecutive signals. **(C)** Left: Representative action potential traces elicited at 1 Hz in default and RA-treated CMs using the perforated patch clamp technique. Right: Quantification of AP-related parameters from independent measurements and cell batches. ^*^p < 0.05 WT+RA vs. WT and KCNA5^fs/fs^. #*p* < 0.05 WT+RA vs. KCNA5^fs/fs^+RA, ^**^p < 0.05 KCNA5^fs/fs^+RA vs. WT and KCNA5^fs/fs^. APD, action potential duration at 20, 50, and 90% or repolarization, RMP, resting membrane potential, APA, maxiumum AP amplitude, Plateau, potential 20 ms after the AP upstroke, dV/dt, maximal upstroke velocity. **(D)** Frequency-dependence of APD_50_. ^*^p < 0.05 WT+RA vs. WT and KCNA5^fs/fs^. #*p* < 0.05 WT+RA vs. KCNA5^fs/fs^+RA, ^**^p < 0.05 KCNA5^fs/fs^+RA vs. WT and KCNA5^fs/fs^.

Electrophysiological analyses on multielectrode arrays (MEAs) revealed that spontaneous beating frequencies were increased in atrial CMs as compared to ventricular ones, as expected given shorter AP durations (APDs) in the former (Figure 3A). In ventricular CMs, beat-to-beat (RR) intervals were indistinguishable between WT and KCNA5^fs/fs^ cells. In atrial-enriched cell preparations, however, KCNA5^fs/fs^ CMs showed slower beating frequencies than isogenic WT controls implying that K_v_1.5 functionally influences atrial CM beat rates (Figure 3A, Videos S2, S3). Analysis of frequency-corrected field potential durations (QTc_max_ intervals, FPDs) likewise showed no difference between the cell lines under default differentiation conditions. Atrial-enriched WT CMs displayed greatly reduced (< 100 ms) QTc_max_ intervals as compared to ventricular WT CMs, which is expected given the lack of a plateau phase in underlying atrial APs. Compared to atrial WT cells, atrial KCNA5^fs/fs^ CMs however showed a substantial FPD prolongation (~200 ms, Figure 3B).

In agreement with these data based on bulk cultures, perforated patch clamp measurements revealed an atrial-specific phenotype of our model at single-cell level. Compared with ventricular-like CMs, APs of RA-treated cells were in general characterized by fast phase-1 repolarization resulting in short APs with a negative plateau phase (Figure 3C). APs in atrial KCNA5^fs/fs^ CMs, however, were significantly prolonged at 20 and 50% of repolarization and had a more positive AP plateau phase, as compared to atrial WT CMs (Figure 3C). Notably, these marked differences in atrial APDs remained conserved at various stimulation frequencies (Figure 3D). By contrast, APDs in ventricular-enriched cultures did not differ significantly between the WT and KCNA5^fs/fs^ CMs. Furthermore, there was no marked difference in AP amplitudes, plateau, or upstroke velocities between the groups (Figure 3C). These data indicate the functional importance of K_v_1.5 in shaping the atrial action potential in the hPSC system and reveal a pronounced cardiac subtype-specific phenotype in the disease model.

**Figure 4:**
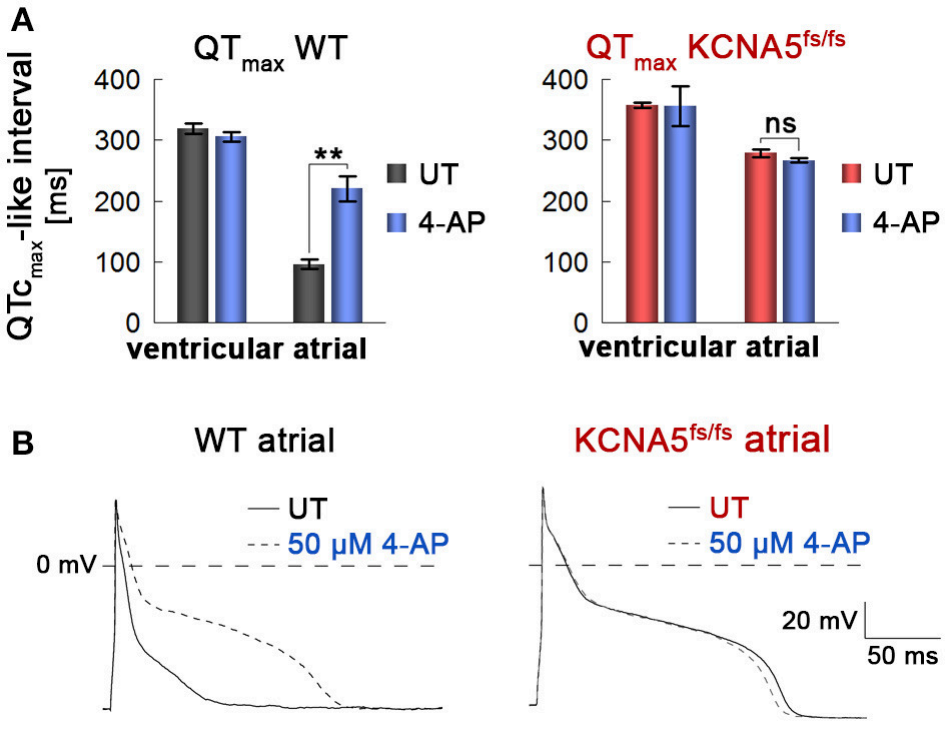
Atrial and WT-specific response to a K_v_1.5 channel blocker. **(A)** Quantification of field potential durations in response to 4-aminopyridine (4-AP, 1 mM, *n* = 3). Note the lack of response in atrial KCNA5^fs/fs^ hiPSC-CMs. ^**^p < 0.01. **(B)** Representative action potential traces at 1 Hz highlighting a specific 4-AP (50 μM) response exclusively in atrial WT CMs.

To affirm that this primary phenotype is indeed due to the loss of K_v_1.5 in atrial KCNA5^fs/fs^ CMs, we next evaluated the effects of 4-aminopyridine (4-AP), an inhibitor of K_v_1.5 (Devalla et al., 2015). 4-AP administration had little effect on beating frequencies (Figure S3A). Likewise, ventricular WT and KCNA5^fs/fs^ CMs did virtually not respond to this compound when assessing FPDs on MEAs or APDs using single-cell patch clamping (Figure 4A, and data not shown). By contrast, 4-AP provoked a significant FPD and APD prolongation specifically in atrial WT cardiomyocytes but exerted no effects in K_v_1.5-deficient KCNA5^fs/fs^ mutants (Figures 4A,B, Figure S3B, Table S1). Taken together, these data imply that the atrial-specific FPD and APD prolongation phenotype in the KCNA5^fs/fs^ model is indeed due to the loss of K_v_1.5 function and that this channel accounts for much of the repolarization reserve.

### KCNA5^fs/fs^-specific drug responses

A patient-specific *KCNA5* nonsense mutation has previously been shown to increase vulnerability for triggered activity under adrenergic stress conditions (Olson et al., 2006). Hence, to assess the relevance of the KCNA5^fs/fs^ model in the context of AF, we exposed atrial WT and mutant CMs to several distinct stimuli and channel blockers. Modulating β-adrenergic signaling using propranolol and isoprenaline promoted a marked decrease and increase in spontaneous beating frequencies, respectively, albeit without causing any irregularities (Figures S4A,B). Furthermore, to interfere with the function of K^+^ channels potentially collaborating with K_v_1.5 in atrial CMs, we employed AF-DX-116, a muscarinic receptor antagonist (to indirectly compromise K_ir_3.1/3.4) (Hammer et al., 1986), and cisapride, a potent hERG blocker (Rampe et al., 1997). These treatments did however not reveal any signs of irregular beating in atrial KCNA5^fs/fs^ CMs (Figures S4C,D), contrasting with our previous results based on ventricular-enriched K_v_7.1-deficient hiPSC-CMs (Zhang et al., 2014).

**Figure 5:**
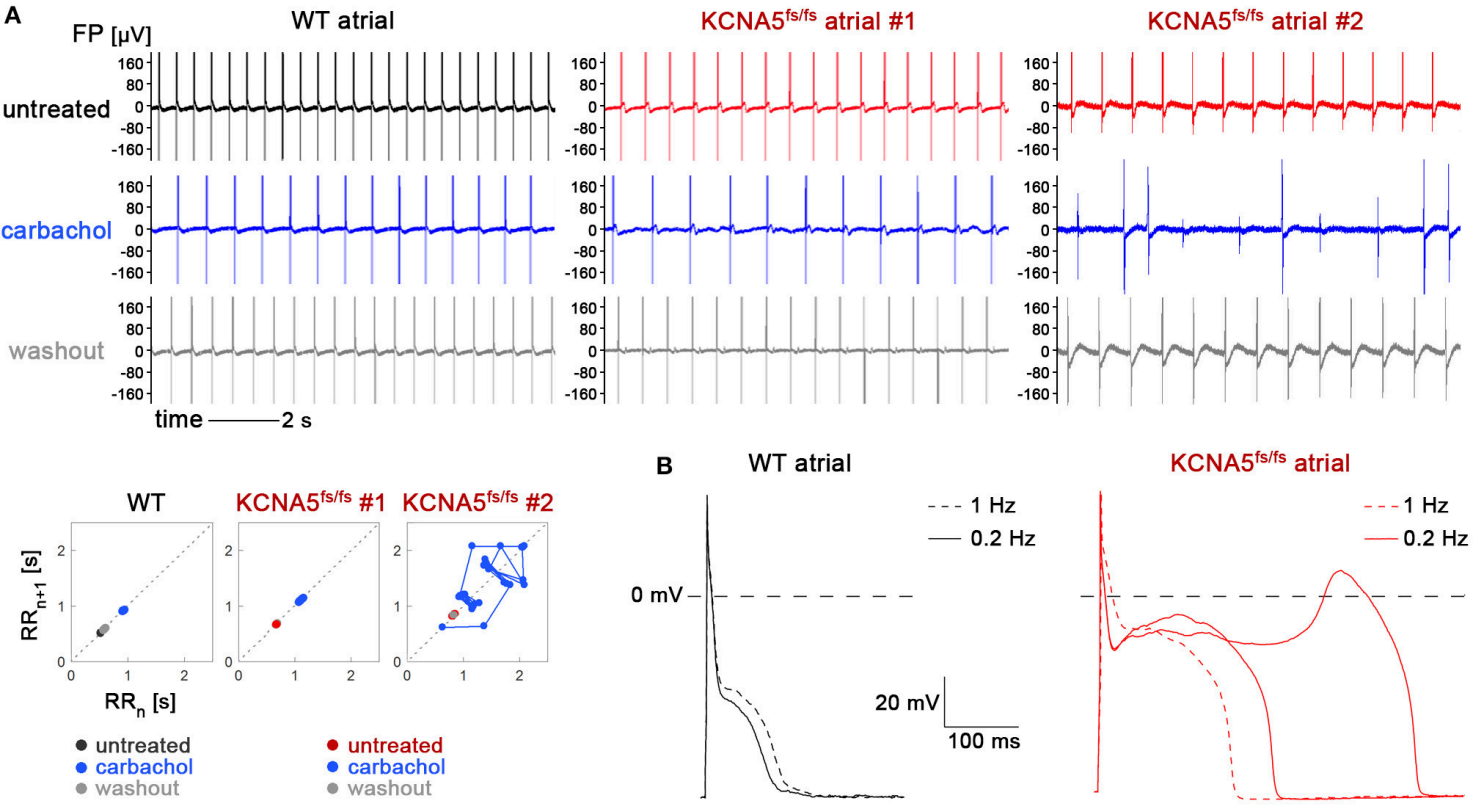
KCNA5^fs/fs^-specific irregularities in spontaneous beating and action potential firing at low stimulation frequency. **(A)** Effects of carbachol stimulation on atrial WT and KCNA5^fs/fs^ hiPSC-CMs. Representative MEA recordings (top) and corresponding Poincaré plots (bottom). Carbachol-induced baseline irregularities (top middle panel) or chaotic beating (top right) was exclusive to atrial KCNA5^fs/fs^ CMs and observed in 4 out of 8 experiments. **(B)** At single-cell level, atrial KCNA5^fs/fs^ CMs were prone to develop EADs at low (0.2 Hz) stimulation frequencies, as observed in 5 out of 12 cells (right). WT controls (left) did not show any action potential irregularities (0 out of 9 cells).

To challenge the model in an alternative manner, we next used carbachol, a cholinergic agonist promoting the acethylcholine-sensitive K^+^ current (I_KACh_). Although this type of treatment might seem counterintuitive in the context of a *KCNA5* mutation, carbachol administration presents an accepted means of inducing AF in animal models (Kovoor et al., 2001). Supplementation of as much as 0.5 mM carbachol to bulk cultures only promoted an expected reduction in beat-to-beat intervals of atrial WT CMs, with no irregularities (Figure 5A, left). By contrast, atrial KCNA5^fs/fs^ CMs tended to show irregular baselines in-between contractions following carbachol administration (Figure 5A, middle), or chaotic beating (Figure 5A, right), which was never seen in WT or ventricular-enriched preparations. These effects were reversible, as normal beating was restored following drug washout, albeit these observations were only made in a subset of experiments (4 out of 8). Because irregular beating in atrial KCNA5^fs/fs^ CMs tended to occur at reduced beating rates (Figure 5A), we finally sought to identify possible triggers for this mutant-specific behavior using patch clamp methodology in single cells at low stimulus frequencies. Interestingly, at 0.2 but not at 1 Hz, pronounced EADs were obtained in atrial KCNA5^fs/fs^ CMs but not in WT ones (Figure 5B). Again, these events were observed in a subset of samples (5 out of 12 cells analyzed). Together, these data show that atrial KCNA5^fs/fs^ hiPSC-CMs are prone to develop EAD-like triggering events that may translate into macroscopic beating irregularities in a stochastic manner.

## Discussion

In this study, we have explored the potential of the hiPSC system for cardiac subtype-specific disease modeling. In this context, RA signaling has proven a powerful means of redirecting the cardiac differentiation cell fate from a default ventricular to an atrial-like identity (Devalla et al., 2015). Despite the fact that this small molecule-based approach does not yield 100% atrial cells and that the default protocol does likewise not fully avoid atrial contaminants, the purities obtained were sufficient to investigate subtype-specific disease phenotypes without need for genetic selection. Indeed, the KCNA5^fs/fs^-specific FPD and APD prolongation phenotypes, as well as the lack of response to 4-AP, were strictly linked to an atrial cell fate obtained via cardiac subtype-specific differentiation. Hence, as to devising functional drug screening paradigms aiming at a higher throughput—which will require specific but robust cell production pipelines—these are encouraging data.

AF is a complex disease that frequently develops secondary to other cardiac conditions and may involve specific organ-linked structures like the pulmonary veins (Schotten et al., 2011). It is hard to think of a faithful means of modeling such contexts with hiPSCs alone, at the present state of the technology (Goedel et al., 2017). By contrast, investigating familial forms of AF in principle bears the potential to reveal aspects or triggers of the disease in a cell autonomous manner, i.e., using hiPSC-CMs *per se*. Mutations in *KCNA5* present a clearcut class of familial AF cases and fortunately, the gene is well-expressed in atrial-enriched hPSC-CMs (Olson et al., 2006; Yang et al., 2009; Christophersen et al., 2013; Hayashi et al., 2015). Since both gain and loss-of-function mutations in *KCNA5* can cause AF, it may be postulated that this must be based on distinct mechanisms. A gain of *KCNA5* function is thought to promote AF through electrical reentry (Schmitt et al., 2014). By contrast, our data shows that a loss of *KCNA5* function in atrial human hiPSC-CMs causes a pronounced prolongation of the action potential and this may promote EADs which are key triggers for AF (Schotten et al., 2011; Christophersen and Ellinor, 2016).

Interestingly, we could stochastically observe EADs in atrial *KCNA5*^fs/fs^ CMs, but never in isogenic wild-type or ventricular cells. This was confined to bradycardic conditions, i.e., low stimulation frequencies in our setting. The strict mutation dependency of these observations strongly suggests a cell autonomous cause of EADs in this K_v_1.5-deficient scenario. Our finding is consistent with those of Olson et al. (Olson et al., 2006) where EADs were observed at low stimulus frequencies in freshly isolated human atrial CMs during I_Kur_ block. We speculate that the EADs may macroscopically translate into irregularities such as chaotic spontaneous beating in the hiPSC system.

Overactivation of I_KACh_ by a gain-of-function mutation in the *GNB2* gene leads to bradycardia by reduction of pacemaker function in humans, as recently shown (Stallmeyer et al., 2017). I_KACh_ is mediated by Kir3.1/Kir3.4 channel subunits that are highly enriched in human sinus node and atria (Gaborit et al., 2007). Consistently and presumably via I_KACh_ activation, our data shows that bradycardia can be induced by carbachol treatment of atrial iPSC-CMs suggesting that this system would also be suited to study I_KACh_ function. Moreover, we here find that administration of a cholinomimetic compound can prime atrial *KCNA5*^fs/fs^ CMs for showing arrhythmia-like beating behavior, which presumably relies on a concomitant decrease in beating frequencies and stochastic EADs as an underlying triggering event.

It is beyond doubt that AF causes are highly diverse. Nonetheless, specific mutations underlying familial AF bear the potential of recapitulating aspects of the disease using patient-derived or engineered hiPSC-CMs in isolation. Moreover, there is a chance that familial AF models may also prove useful in more general, for devising functional correction assays in drug screening paradigms, for instance. Our study provides a first hiPSC model in this context and strongly encourages efforts to generate additional models of familial AF, such as hiPSC lines carrying *KCNA5* gain-of-function mutations as a next step.

In sum, we have used a combination of cardiac subtype-specific hiPSC differentiation and targeted genetic manipulation to generate a *KCNA5*^fs/fs^ disease model that recapitulates clinical features of K_v_1.5 deficiency. Furthermore, beyond primary phenotypes associated with the loss of K_v_1.5 function, our data encourage the idea that familial AF models may be utilized for investigating triggers of the disease at the cellular level as well as for devising assays aiming at drug-mediated phenotype correction. This exciting perspective, however, will require macroscopic readouts that would indicate disease-specific irregularities in all mutant samples/cell preparations rather than merely in a stochastic fraction of these. This requirement of compatibility with functional screening paradigms hence calls for more AF-specific and potentially more reliable readouts compared with monitoring spontaneous beat rhythm abnormalities. For instance, it will be interesting in future investigation to combine hiPSC-based genetic AF models with optical or phase-mapping methodologies enabling the detection of triggered activity or spiral wave circuitries at the macroscopic level (Umapathy et al., 2010; Bingen et al., 2014).

## Author contributions

AV and BG designed experiments. MM, IP, IM, JF, AR, AV, and BG performed experiments. MM, IP, GS, AV, and BG analyzed the data. GS, AV, and BG wrote the manuscript with input from co-authors.

### Conflict of interest statement

The authors declare that the research was conducted in the absence of any commercial or financial relationships that could be construed as a potential conflict of interest.

## Supplementary material

Supplementary Figure 1 Generation and differentiation of F1 hiPSCs. **(A)** Left: Reprogramming factor expression from polycistronic self-replicating RNA vector in freshly transfected parental fibroblasts (RT-qPCR data, n = 2). Right: Immunostaining prior to enriching the reprogramming factorexpressing fraction further by puromycin selection. **(C)** Embryoid body-based spontaneous differentiation of F1 hiPSCs into derivatives of the three germ layers, following EB attachment and outgrowth (3 wk). **(C)** Optimization of directed cardiac differentiation using F1 hiPSCs by varying the initial BMP4 stimulation dose, while keeping other signaling factor concentrations at constant standard levels. Left: FACS analysis (n = 3). Right: Immunostaining of replated CMs (< 2 wk). Later in this study, the non-CM fraction appeared to be further reduced.

Supplementary Figure 2 Optimization of cardiac subtype-specific directed differentiation. **(A)** Titration of retinoic acid concentration applied on days 3-4 of differentiation (RT-qPCR analysis, n = 4). An RA dose of 0.5 μg/ml was sufficient to almost fully suppress ventricular gene expression in favor of maximum atrial marker induction including KCNA5. Overall pan-cardiac markers appeared to be somewhat compromised in primary differentiation cultures analyzed in these experiments but tended to recover following replating of the cells. **(B)** Immunostaining of replated WT and KCNA5fs/fs CMs confirming this fact and showing *K*_v_1.5 expression exlusively in atrial WT CMs.

Supplementary Figure 3 CM subtype-specific 4-AP responses. **(A)** Beat-to-beat intervals were only moderately affected by 4-AP in WT and KCNA5fs/fs hiPSC-cardiomyocytes, regardless of the CM subtype (n = 3). **(B)** Representative MEA traces of untreated (top) and *K*_v_1.5 inhibitor-treated CMs (bottom). Note the comparatively short QTmax interval in atrial WT cells and the pronounced relative prolongation by 4- AP exclusively in this sample type.

Supplementary Figure 4 Invariant drug responses between atrial WT and KCNA5fs/fs hiPSC-CMs. **(A)** Propranolol causes beat frequency reduction without promoting beat-to-beat variability. **(B)** Isoprenaline accelerates spontaneous beating without provoking arrhthmia. **(C)** M2 muscarinic receptor antagonist AF-DX-116 promotes a slight increase in beating frequencies of atrial WT and KCNA5fs/fs hiPSC-CMs. **(D)** hERG channel blocker cisapride does not affect beating behavior in atrial hiPSC-CMs.

Table S1 Quantification of AP parameters in RA-treated WT (*n* = 5) and KCNA5^fs/fs^ (*n* = 6) CMs with and without 4-AP treatment.

Table S2 Oligonucleotides used in this study.

Video S1 Primary monolayer of F1 hiPSC-CMs at day 9 of directed cardiac differentiation.

Video S2 Replated WT CMs differentiated into an atrial-like fate via transient RA supplementation.

Video S3 Replated atrial KCNA5^fs/fs^ CMs.
