# Supplementary material for: Cardiac Subtype-Specific Modeling of Kv1.5 Ion Channel Deficiency Using Human Pluripotent Stem Cells
Source: Front Physiol. 2017 Jul 6;8:469. doi: 10.3389/fphys.2017.00469 (PMC5498524; doi:10.3389/fphys.2017.00469)
Supplement: Supplementary Figure 1 — Generation and differentiation of F1 hiPSCs. (A) Left: Reprogramming factor expression from polycistronic self-replicating RNA vector in freshly transfected parental fibroblasts (RT-qPCR data, n = 2). Right: Immunostaining prior to enriching the reprogramming factorexpressing fraction further by puromycin selection. (C) Embryoid body-based spontaneous differentiation of F1 hiPSCs into derivatives of the three germ layers, following EB attachment and outgrowth (3 wk). (C) Optimization of directed cardiac differentiation using F1 hiPSCs by varying the initial BMP4 stimulation dose, while keeping other signaling factor concentrations at constant standard levels. Left: FACS analysis (n = 3). Right: Immunostaining of replated CMs (< 2 wk). Later in this study, the non-CM fraction appeared to be further reduced. [file SupplementaryFigures.PDF]

SUPPLEMENTARY FIGURES

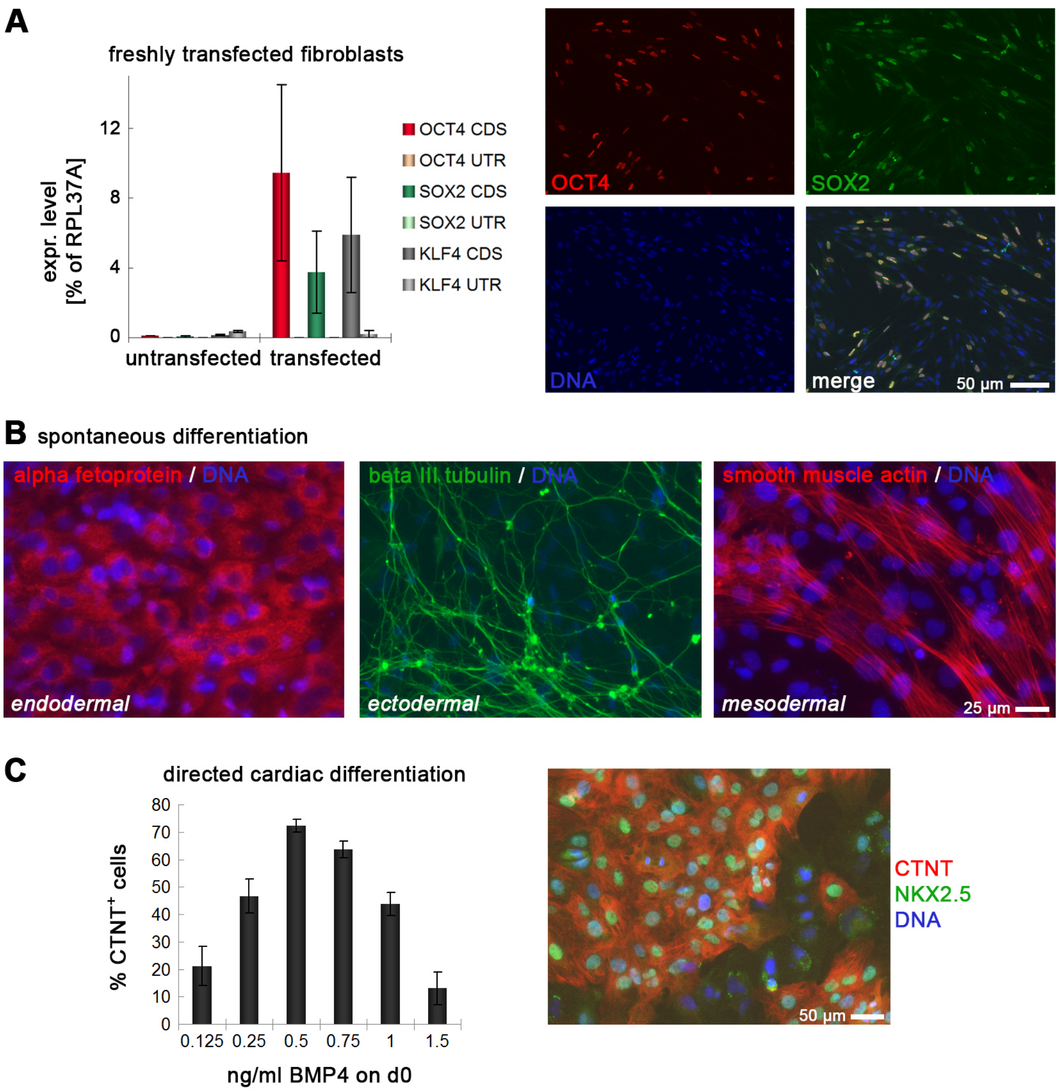

FIGURE S1 Generation and differentiation of F1 hiPSCs. (A) Left: Reprogramming factor expression from polycistronic self-replicating RNA vector in freshly transfected parental fibroblasts (RT-qPCR data, n = 2). Right: Immunostaining prior to enriching the reprogramming factor-expressing fraction further by puromycin selection. **(C)** Embryoid body-based spontaneous differentiation of F1 hiPSCs into derivatives of the three germ layers, following EB attachment and outgrowth (~3 wk). **(C)** Optimization of directed cardiac differentiation using F1 hiPSCs by varying the initial BMP4 stimulation dose, while keeping other signaling factor concentrations at constant standard levels. Left: FACS analysis (n = 3). Right: Immunostaining of replated CMs (< 2 wk). Later in this study, the non-CM fraction appeared to be further reduced.

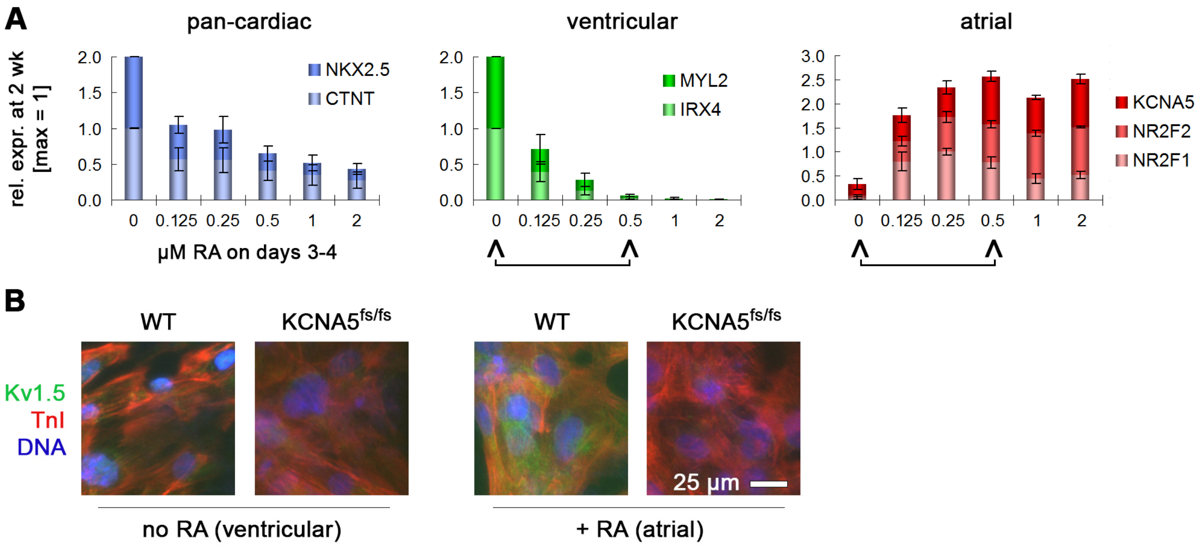

FIGURE S2 Optimization of cardiac subtype-specific directed differentiation. (A) Titration of retinoic acid concentration applied on days 3-4 of differentiation (RT-qPCR analysis, n = 4). An RA dose of 0.5 µg/ml was sufficient to almost fully suppress ventricular gene expression in favor of maximum atrial marker induction including *KCNA5*. Overall pan-cardiac markers appeared to be somewhat compromised in primary differentiation cultures analyzed in these experiments but tended to recover following replating of the cells. **(B)** Immunostaining of replated WT and KCNA5$^{fs/fs}$ CMs confirming this fact and showing K_v1.5 expression exlusively in atrial WT CMs.

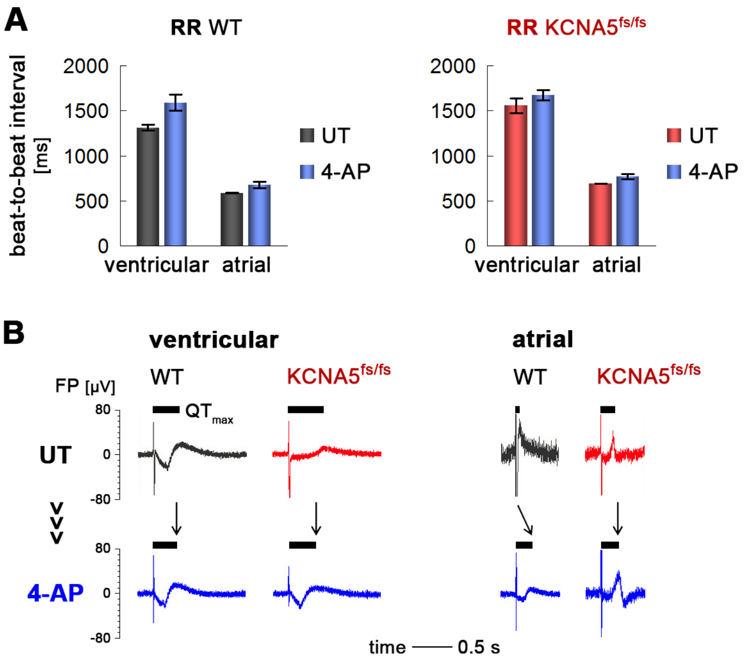

FIGURE S3 CM subtype-specific 4-AP responses. (A) Beat-to-beat intervals were only moderately affected by 4-AP in WT and KCNA5$^{fs/fs}$ hiPSC-cardiomyocytes, regardless of the CM subtype (n = 3). **(B)** Representative MEA traces of untreated (top) and K_v1.5 inhibitor-treated CMs (bottom). Note the comparatively short QT_{max} interval in atrial WT cells and the pronounced relative prolongation by 4-AP exclusively in this sample type.

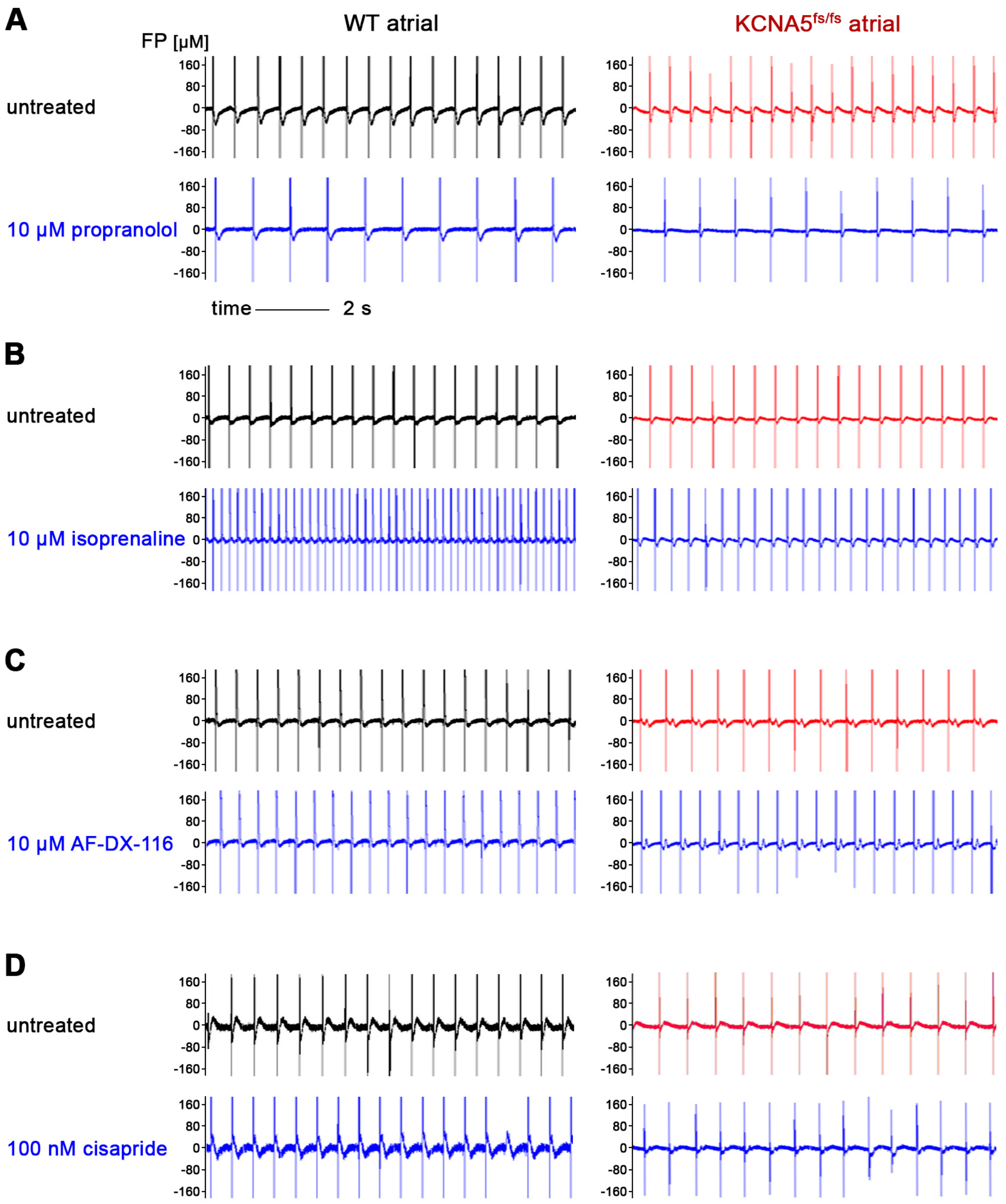

FIGURE S4 Invariant drug responses between atrial WT and KCNA5$^{fs/fs}$ hiPSC-CMs. (A) Propranolol causes beat frequency reduction without promoting beat-to-beat variability. **(B)** Isoprenaline accelerates spontaneous beating without provoking arrhythmia. **(C)** M2 muscarinic receptor antagonist AF-DX-116 promotes a slight increase in beating frequencies of atrial WT and KCNA5$^{fs/fs}$ hiPSC-CMs. **(D)** hERG channel blocker cisapride does not affect beating behavior in atrial hiPSC-CMs.
